# Process and experience of youth researchers within a Health Promoting Schools study in Nova Scotia, Canada

**DOI:** 10.1093/heapro/daad174

**Published:** 2023-12-20

**Authors:** Julia C Kontak, Hilary A T Caldwell, Rena Kulczycki, Camille L Hancock Friesen, Sara F L Kirk

**Affiliations:** Faculty of Health, Dalhousie University, 5968 College Street, PO Box 15000, Halifax, Nova Scotia, B3H 4R2, Canada; Healthy Populations, Institute, Faculty of Health, Dalhousie University, 1318 Robie Street, Halifax, NS, B3H 3E2, Canada; Healthy Populations, Institute, Faculty of Health, Dalhousie University, 1318 Robie Street, Halifax, NS, B3H 3E2, Canada; Healthy Populations, Institute, Faculty of Health, Dalhousie University, 1318 Robie Street, Halifax, NS, B3H 3E2, Canada; Healthy Populations, Institute, Faculty of Health, Dalhousie University, 1318 Robie Street, Halifax, NS, B3H 3E2, Canada; Division of Pediatric Cardiothoracic Surgery, Children’s Nebraska, University of Nebraska Medical Center, 8200 Dodge Street, Omaha, NE 68114, USA; Healthy Populations, Institute, Faculty of Health, Dalhousie University, 1318 Robie Street, Halifax, NS, B3H 3E2, Canada; School of Health and Human Performance, Faculty of Health, Dalhousie University, PO Box 15000, Halifax, NS B3H 4R2, Canada

**Keywords:** student engagement, comprehensive school health, school health, participatory action research

## Abstract

Youth Participatory Action Research (YPAR) is an approach to research that engages youth across the research process. The peer researcher method is a technique used in YPAR where youth are trained in research and ethics to interview their peers. The purpose of this study was to: (i) describe the process of engaging youth as peer researchers in a Health Promoting Schools (HPS) and student engagement project and (ii) understand the peer researchers’ perspectives of their experience throughout the project. Youth from across Nova Scotia, Canada in grades 7–10 (ages 12–16) were recruited as peer researchers in the Summer, 2022. The project included three stages: (i) peer researcher training, (ii) practicing, recruiting and conducting interviews and (iii) data interpretation workshop. To understand the peer researcher’s experience, quantitative data were collected from an evaluation questionnaire. Outputs were produced using descriptive statistics. Qualitative data were collected through a focus group and interviews and analyzed using inductive content analysis. A total of 11 youth were recruited and completed peer researcher training. Most youth provided positive feedback on the training with a satisfaction score of 8.7/10. Qualitative analysis indicated benefits to the peer researchers including opportunities to build interview and social skills and learn about other’s perspectives. This study provides a detailed overview of how to use a peer researcher method in a YPAR project to involve youth in research related to HPS and student engagement. The research also highlights the benefits of engaging youth in YPAR. Future research will report on the findings from the peer interviews.

## Graphical Abstract

**Graphical Abstract:**
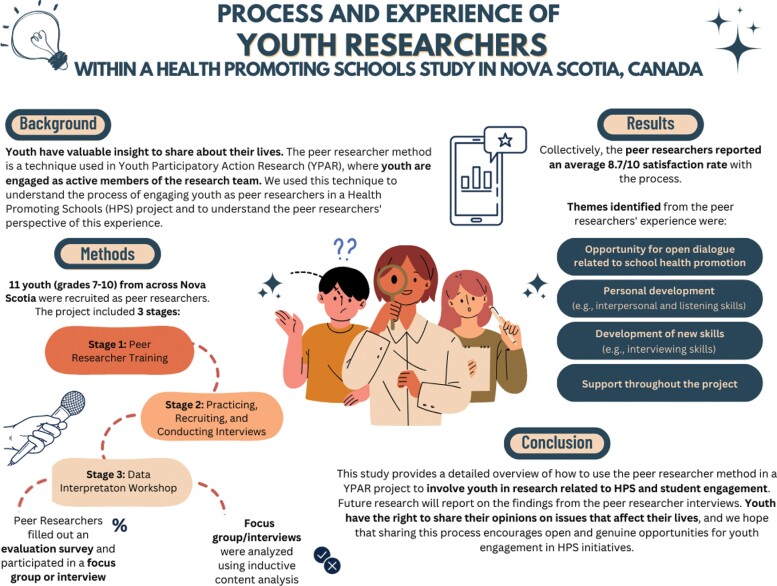

**Contribution to Health Promotion**

- The article provides a useful and practical overview of how to meaningfully engage youth in various stages of a school health promotion research project.
- This research provides insight into the benefits of engaging youth in school health promotion research including building interview and social skills and learning from other’s perspectives.
- This work contributes to the growing school health promotion effort to authentically engage students in decision-making practices.

## INTRODUCTION

There is a growing demand for strategies to engage youth actively and meaningfully in health issues of concern since the ratification of the United Nations 1989 Convention on the Rights of the Child (United Nations, 1989). Of note, is the principle of participation in Article 12 highlighting that ‘children have the right to give their opinions in all matters that affect them and to have their voices heard’ (United Nations, 1989). A participatory action research (PAR) approach with younger people is readily being used to facilitate and encourage youth participation in systematic research projects (Powers & Tiffany, 2006; Lawson et al., 2015; Kim, 2016; Ozer, 2017). Known generally as Youth Participatory Action Research (hereon referred to as YPAR), is an approach to conducting research *with*, rather than *on* youth (Powers and Tiffany, 2006; Lawson *et al.*, 2015). YPAR aims to engage youth as *active* members throughout different phases of the research process through youth–adult partnerships, rather than simply participants (Powers & Tiffany, 2006; Lawson et al., 2015; Kim, 2016). According to Rodríguez and Brown (2009), there are three guiding principles to YPAR: (i) it is inquiry-based—grounded in youth experiences on an issue; (ii) it is participatory—youth are collaborators throughout the research process and (iii) it is transformative—intervenes to change knowledge practices and share power between youth and adults. However, as conceptual principles of YPAR focus more on *who* is involved throughout the research process, and *why* rather than the specific set of methods implemented (Bergold & Thomas, 2012; Caraballo et al., 2017), there is a lack of dedicated reporting on the methodological techniques used in YPAR and how they are conducted in practice. This gap is specifically evident in the field of school health promotion.

### Youth Participatory Action Research

Participatory approaches first stemmed from philosophical views developed in opposition to dominant research paradigms that situate researchers as the sole proprietors of knowledge (Rodríguez & Brown, 2009; Creswell & Poth, 2016). YPAR disrupts traditional ways of knowing by adopting a transformative paradigmatic way of thinking. A transformative paradigm is grounded in collaborative research processes, encourages multiple ways of knowing, highlights health and equity issues of concern and dismantles power dynamics between adults and youth partners (Caraballo et al., 2017).

YPAR offers benefits at the individual, interpersonal and system level. For youth, benefits include increased agency, leadership, social and interpersonal skills and improved health and cognitive behaviours (Ozer, 2017; Anyon et al., 2018; Branquinho et al., 2020). For communities and organizations, YPAR can improve understanding of youth issues, enhance relationships with young adults and help shift organizational cultures to be more inclusive of youth voice (London et al., 2003; Shamrova & Cummings, 2017; Branquinho et al., 2020).

Beyond human and organizational benefits, the methods employed in YPAR play a critical role in enhancing the scientific study of issues affecting young people’s lives. Engaging youth in various stages of the research process including data collection, analysis and interpretation of data (Powers & Tiffany, 2006; Ozer, 2017) helps to mitigate power dynamics (Kim, 2016), enhance authentic participation (Garnett et al., 2019; Kennedy et al., 2019; Sprague Martinez et al., 2020) and provide value and applicability to the research findings (Ozer, 2017). Common methods used in YPAR include photovoice (Wang & Burris, 1997), a visual method that gathers evidence through youth researchers taking photographs of the phenomenon under inquiry (Wang & Burris, 1997), as well as the draw-and-write technique that involves asking a young person to draw a picture in response to a research question and to write down any comments associated with the ideas (Lima & Lemos, 2014). Yet, a novel, and understudied technique, is the peer researcher method (Lile & Richards, 2018). This is where youth engage in research and ethics training to establish their competence in conducting interviews and/or focus groups with their peers (Lile & Richards, 2018; Kontak et al., 2022). The peer researcher method moves away from conventional techniques of adults conducting interviews on youth (Lile & Richards, 2018; Kontak et al., 2022) and can provide credibility to an array of methodological components, including feasibility of recruitment, appropriateness of interview tools, feedback on data collection techniques, quality of data collected and the meaningfulness of the research findings (Hawke et al., 2018, p. 20; Jardine & James, 2012).

### School health promotion

YPAR approaches have shown promise as a useful approach to gathering student views on specific school health promotion issues (Powers & Tiffany, 2006; Jennifer, 2014; Anselma et al., 2020), including the use of peer research methods on issues related to food and nutrition (Lile & Richards, 2018; Browne et al., 2020) and tobacco use (Jardine & James, 2012; Nelson Ferguson et al., 2023). School health promotion is any activity, project, program or initiative that aims to promote health, or other social and environmental determinants that impact the health of students or school–community members (Griebler et al., 2017). Health Promoting Schools (HPS) is a whole-school approach that consistently ‘strengthens the capacity of schools as safe and healthy settings for living, learning and working’ (World Health Organization [WHO], 2021, p. 1). HPS is widely adopted across the globe and is in alignment with the United Nations Convention on the Rights of the Child (United Nations, 1989). An essential tenet of HPS is the meaningful involvement of youth in school health promotion. However, this commitment may be impeded as school health promotion policies, and programs are often pre-determined without the perspectives and opinions of youth being considered (Anselma et al., 2020). YPAR and related methods are valuable and effective in ensuring students are actively engaged in developing and making decisions related to school health promotion.

### Local context and purpose

The HPS model has been implemented in Nova Scotia, Canada since 2005, yet research suggests there is a lack of youth engagement within the current provincial model (McIsaac et al., 2015, 2017; Kontak et al., 2022). Recently, UpLift, a school–community partnership was developed to help catalyze HPS in the province, with an emphasis on increased youth engagement. One of several strategies to increase youth engagement in HPS was the development and implementation of a YPAR project using a peer researcher method. Youth from across Nova Scotia came together to be trained as peer researchers through building skills in interview methods and ethics as well as gathering perspectives from their peers on youth engagement and health promotion within the school context. This project was completed twice (2021 and 2022) with two different groups of peer researchers and interview participants. The initial findings from the first iteration of interviews are published in *Health Promotion International* (Kontak et al., 2022). In the first round of interviews, participants highlighted the importance of the social and physical school environments on their health and well-being, including the significance of a supportive and safe environment. Participants also indicated numerous factors impacting student engagement including inclusive practices such as accessibility, awareness and safe spaces, having an interest in the topic and their voice being heard, as well as power dynamics between students and adults. Building on that work, the purpose of this article is to: (i) provide a detailed summary of the use of the peer researcher method in the YPAR approach and (ii) outline the evaluation findings based on participant experiences. The information in this article is from the second round of the YPAR project where an outcome evaluation was conducted after the peer researcher method was completed. Future research will report on the findings from the peer interviews.

## METHODS

### Philosophical paradigm

This study adopted a transformative paradigm to the research recognizing the importance of collaborative research, the active dismantling of power differentials, the recognition of multiple ways of knowing and the importance of advocating for social and equity issues within an agenda for change (Mertens, 2010; Creswell & Poth, 2016). With a specific focus on youth in this project, the epistemological assumption of a transformative paradigm specifically addresses the imbalance of power and privilege by working to shift, transform and redefine knowledge through engaging the voice of participants who are often excluded from positions of power (Mertens, 2010).

### Researchers’ position

The researchers involved in this project hold the underlying theoretical belief and practical conception of the integral importance of engaging young people in research and practice that affect their lives. All researchers work in an academic or practical setting that aims to amplify the voice of young people, specifically in relation to school health and well-being. The researchers acknowledge that their understanding of youth engagement and qualitative design has evolved over time and that they continue to learn and relearn how historical structures and systemic issues significantly impact definitions and interpretations of power and knowledge.

### Recruitment and participants

Convenience sampling methods (Creswell and Poth, 2016) were used to recruit peer researchers for the YPAR project in June of 2022. Members of the research team recruited peer researchers for this training by reaching out to adults who work directly with youth on student-led school health promotion initiatives, youth-serving organizations and key contacts, in and across school regions of Nova Scotia. Detailed explanations of the training were provided to contacts, including a promotional poster describing the purpose of the research and the role of the youth as peer researchers. The inclusion criteria for participating were students either going into or graduating from grades 7–10 (ages 12–16) in a public school in Nova Scotia, Canada. Once a youth and/or a guardian expressed interest, communication occurred directly with them through a phone or virtual meeting to explain and outline the project. If youth were interested in the project, a consent form, photo release form and form for compensation were sent to the participant and their guardian to review and sign prior to participation.

### Youth Participatory Action Research (YPAR) project

**Fig. 1:**
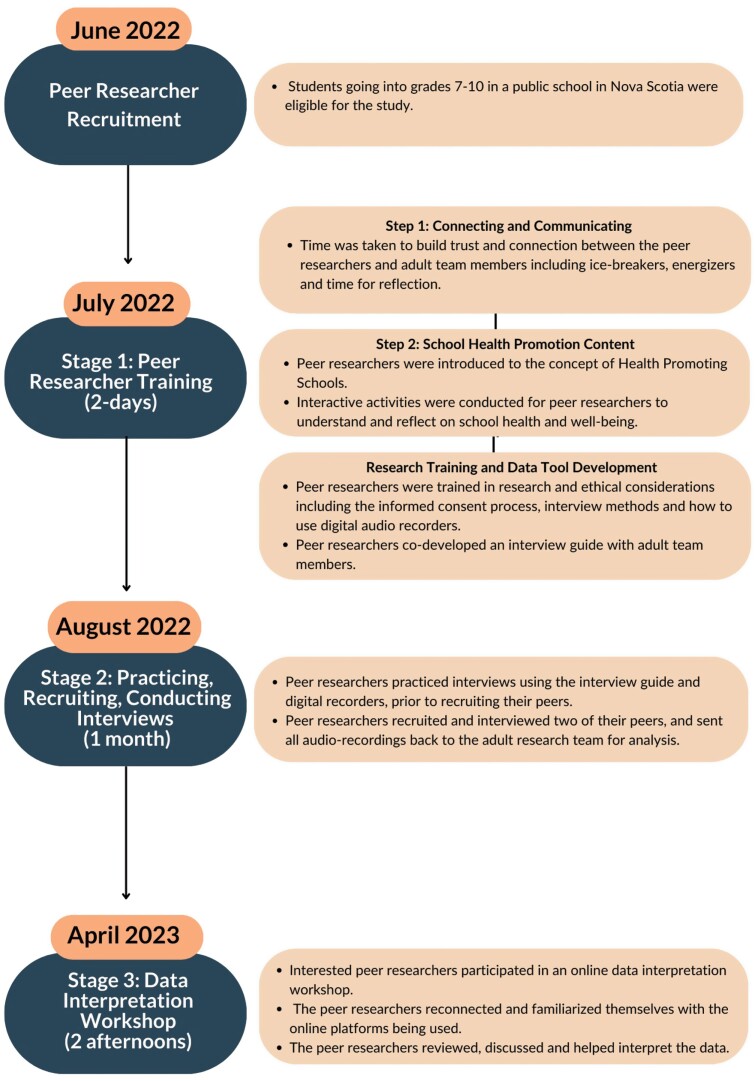
Stages and timeline of the YPAR project.

The YPAR project consisted of three stages: (i) peer researcher training, (ii) practicing, recruiting and conducting interviews and (iii) data interpretation workshop. Stages 1 and 2 took place in Summer 2022 (July and August) and stage 3 (data interpretation workshop) was completed in April of 2023, after the interview findings were initially analyzed by the lead author (J.C.K.). The peer researcher training involved a 2-day, in-person training workshop where youth from across the province came together to connect with other peer researchers, gain an understanding of the purpose and content of the research study and be trained in interview methods and ethics. This training was broken down into three sections: (i) Connecting and Communicating, (ii) School Health Promotion Content and (iii) Research Training. Supplementary Document 1 shows a sample agenda that was adapted based on youth needs during the workshop. Members of the research team worked with an external facilitator who has expertise in youth engagement, school health promotion and participatory research to deliver the training. Each stage of the YPAR project is described in more detail below, while Figure 1 displays the stages visually alongside the timeline of the process.

### Stage 1: Peer researcher training

#### Connecting and communicating

A portion of the training was dedicated to connecting, building trust, cohesion, comfort and communications across the peer researchers and adult facilitators to help foster collaboration and dismantle power dynamics between adults and peer researchers. This involved several facilitated activities including icebreakers and energizer games and building community agreements. Community agreements are a common facilitation practice to decide across a group how they would like to work together. Time for reflection was also a core component of the process, to ensure space was available to discuss and ask questions throughout each part of the training sessions. One example is the rose, bud and thorn reflection activity where peer researchers shared a positive from the day (rose), something they are looking forward to in the training or in their life (bud) and something they needed more support from in the training or in their life (thorn) (see example in Supplementary Document 2).

#### School health promotion content

Peer researchers engaged in content learning and interactive activities, led by the research team, to build their knowledge related to school health promotion. The content included an overview of the basic principles of an HPS approach, including the UpLift partnership operating in Nova Scotia. Of note, specific examples of how students act as change-makers within school health promotion were shared including the description of different student-led projects across the province such as the development of school gardens, smoothie bars and safe spaces for mental health support. These examples were shared as a way for peer researchers to understand their abilities and power to make change within their own school community and beyond. The interactive activity included peer researchers collaborating to describe the concepts of ideal school health and well-being in small group conversations based on sensory prompts such as ‘What does it look like?’, ‘What does it feel like?’, ‘What does it smell like?’ and ‘What are people doing?’. Peer researchers were asked to write down all their answers on a flipchart and share back to the larger group (see example in Supplementary Document 3 ). A group discussion then followed to discuss the reality of school health and well-being within the local school context to build further knowledge and understanding of school health promotion principles.

#### Research training and data tool development

Research training for peer researchers included a presentation by an adult facilitator on the purpose of the research, participatory methods, ethical considerations, how to develop and conduct semi-structured interviews and the roles and responsibilities of the peer researchers in the project. Peer researchers were trained on how to use digital recorders. They then co-developed interview questions related to student engagement and school health and well-being. Peer researchers then used the interview guides and digital recorders to practice with other trainees to develop their skills and comfort in conducting one-on-one semi-structured interviews.

### Stage 2: Practicing, recruiting and conducting interviews

After the 2-day training, peer researchers were provided with a take-home package that included: (i) general information on the project, (ii) the co-developed interview guide, (iii) consent forms for administering interviews, (iv) $15 gift cards for interview participants, (v) time sheets to record their work, (vi) a print out of the research training presentation, (vii) a resource sheet related to mental health support services across the province and (viii) a pre-addressed, prepaid envelope to send documents (i.e. consent forms, time sheet) and digital recorders back to the research team after interviews were completed. Peer researchers were asked to complete two interviews within a 1-month period and were responsible for recruiting peers, administering and collecting signed consent forms from interviewees and their guardians and conducting interviews. Peer researchers were provided written information on how to reach out to the research team for support and if they had any follow-up questions. The adult team members who facilitated stage 1—peer researcher training also reiterated to peer researchers to reach out for guidance at any point if they had any questions or needed help practicing, recruiting and interviewing their peers, such as understanding consent forms, the interview guide, digital recorders and timesheets. As J.C.K. was the one who coordinated the recruitment, their email was provided for any questions or concerns. Only one peer researcher reached out during this time span to ask about assistance with the digital recorders and assistance was provided to the peer researcher by email.

### Stage 3: Data interpretation workshop

Following a preliminary analysis of the interview findings by the first author (J.C.K.), a data interpretation workshop was held online in April 2023. This workshop provided the peer researchers with an opportunity to reconnect with others, review, interpret and provide their feedback on the findings. This workshop was broken up into two online sessions (1.5–2 h in length) to provide an opportunity and time to reconnect, familiarize the peer researchers with the online platforms used (Zoom and Mural), as well as review and discuss the findings of the interviews they conducted. These workshops were facilitated by two members of the research team who also facilitated the peer researcher training (J.C.K. and R.K.), as well as two-note takers to capture peer researchers’ thoughts and perspectives on the interview findings. Peer researchers’ interpretation and feedback will inform the analysis and writing of the interview findings that will be reported separately.

### Materials and data collection

#### Questionnaire

To understand peer researcher satisfaction and learnings throughout the 2-day in-person training workshop, a short evaluation questionnaire comprised of closed- and open-ended questions was completed post stage 1—peer research training. Closed-ended questions were evaluated on a 5-point Likert scale ranging from Strongly Disagree to Strongly Agree. Open-ended questions included fill-in comment boxes, such as *‘What were the best parts of the training and why?’.* The evaluation questionnaire can be reviewed in Supplementary Document 4.

#### Focus group and interviews

To further understand peer researchers’ learning and overall experience, an online focus group was conducted post completion of stage 2—practicing, recruiting and conducting interviews. If peer researchers could not attend the focus group due to scheduling conflicts, a one-on-one online interview was scheduled. The focus group was approximately 1.5 h including time for a check-in and check-out question. A semi-structured interview guide was developed prior to conducting the focus groups/interviews and included questions such as, ‘*What did you learn from the project?*’, ‘*What would you change/improve*?’ and ‘*How can you apply your training in the future?*’. The full interview guide can be reviewed in Supplementary Document 5. Peer researchers were invited to answer the questions orally and/or verbally in the chat function depending on their comfort level. The focus group and interviews were all conducted and recorded on the Zoom virtual platform.

### Ethical considerations

The Social Sciences and Humanities Research Ethics Board at Dalhousie University, Nova Scotia, Canada reviewed and approved the project [file #: 2021-5701]. Consent was obtained from the peer researchers and interviewees, as well as both groups’ guardians. For the peer researchers, guardian consent was obtained prior to peer researcher training.

All peer researchers were compensated for their time at a rate of $13.50/h (Canadian [CAD]), which was slightly above the provincial minimum wage in 2021/2022 ($13.35/h CAD). Overall, the time commitment for the peer researchers was approximately 30 h.

### Data processing and analysis

Questionnaire data were entered into a Microsoft Excel spreadsheet and descriptive data analysis was conducted for quantitative data. Open-ended data from the evaluation questionnaire were imported into Nvivo Qualitative Data Software, version 1.7.1 (Lumivero, 2023) for analysis in conjunction with focus group and interview data.

Audio recordings from the focus group and interviews were transcribed, de-identified and imported into Nvivo Qualitative Data Software (Lumivero, 2023) for analysis. Qualitative description (Sandelowski, 2000) through inductive content analysis (Hsieh & Shannon, 2005) was used to analyze qualitative data. As the research used a transformative paradigm to heighten the voice of the participants, a qualitative descriptive approach was taken, such that the research team wanted to stay close to the data with minimal interpretation of the peer researchers’ perceptions (Sandelowski, 2000). Inductive content analysis was then employed to identify codes and develop themes. All data were saved on a private institutional OneDrive folder and all data analysis was conducted by the lead researcher (J.C.K.).

Multiple trustworthiness techniques to ensure qualitative rigour were implemented: (i) enhancement of credibility through a qualitative descriptive approach, (ii) increased dependability by contextualizing the findings through the in-depth description of the methods, (iii) confirmability through the integration of feedback from the adult team members, (iv) increased transferability based on the diverse group of peer researchers and (v) continual reflexive practices to acknowledge the power and biases of the adult team members.

## RESULTS

Overall 21 youth expressed interest with 11 youth being recruited and consented for peer researcher training in the summer of 2022. The number of peer researchers was capped at 11 due to funding resources and ensuring that there was an appropriate number of participants for an intensive training workshop. Peer researchers were observed to be diverse in race (White presenting, *n* = 5, Black presenting, *n* = 4, Indigenous presenting, *n* = 1 and person of colour presenting, *n* = 1), gender (girl identifying, *n* = 6, boy identifying = 5) and resided in five of the seven geographic school regions in Nova Scotia. All 11 peer researchers completed the evaluation questionnaire, nine attended the online focus group and two completed online interviews as they were unable to attend the focus group. Of the 11 peer researchers, 9 expressed interest in attending and 6 actually attended the data interpretation workshop.

### Quantitative findings

**Fig. 2:**
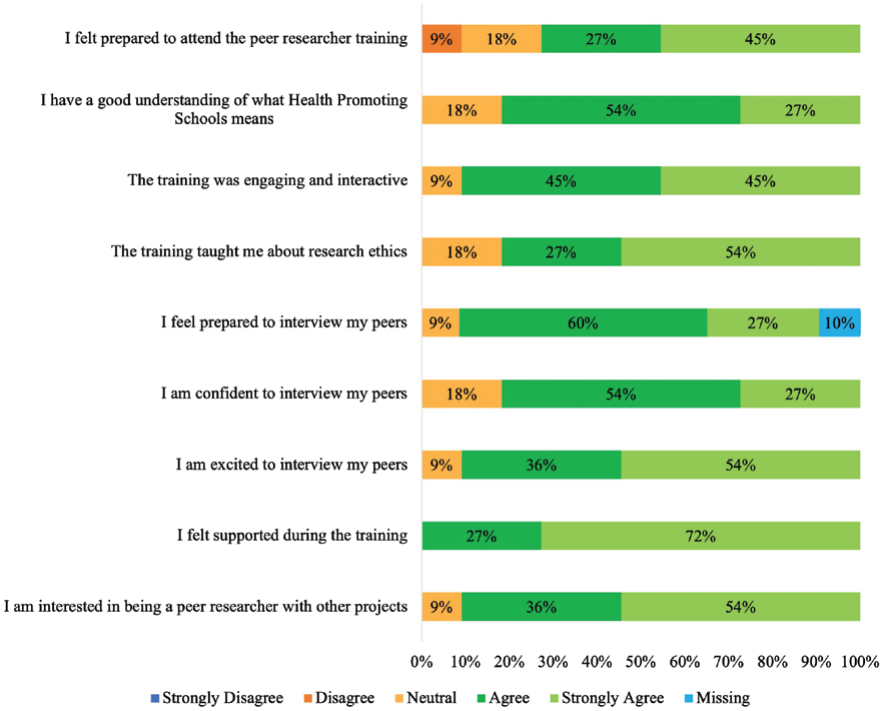
Quantitative evaluation findings from the peer researcher training workshop.

Most peer researchers provided positive feedback on stage 1—peer researcher training of the YPAR project with an average score of 8.7/10 when asked overall how satisfied they were with the training. Most reported they ‘Agreed’ or ‘Strongly Agreed’ with questions asked including, ‘having a good understanding of what HPS means’ (81%, *n* = 9) and ‘the training was engaging and interactive’ (90%, *n* = 10) (Figure 2).

### Qualitative findings

Four themes were developed from the peer researchers’ overall experience of being part of the project, with most of the discussion relating to their learnings, including: (i) development of new skills, (ii) personal development, (iii) opportunity for open dialogue related to school health promotion and (iv) support throughout the project.

#### Development of new skills

Most peer researchers shared that conducting interviews was a new skill they learned, with some reporting it was something they had never done before and would be valuable for future career opportunities. As noted by one participant, *‘I think it was probably the most helpful because I got to learn how to interview in case I ever needed that in the future*’. The skills learned from the interview process ranged in scope from basic skills of giving and taking an interview and the importance of practice, to the ability to learn how to prompt and rephrase questions. However, some peer researchers were not certain of the appropriateness of rephrasing questions and that this was considered a useful skill for qualitative data collection. As shared by one participant, *‘…I’d paused, I paused the recorder…I’d say something like…a similar question, or something. Or… if I already knew something about their school, or the school that we share together…I’d say something that it might involve, so then they could get like an idea of it’.*

#### Personal development

Peer researchers identified that the process of interviewing helped build and enhance personal development skills. Of note, the training helped peer researchers build interpersonal skills, minimize nerves and get them out of their *‘shell’* through meeting new people, conversing with peers, practicing and conducting interviews. As shared by one individual, ‘*Meeting new people and talking outwardly helped with my social anxiety’.* Peer researchers also named that the process enhanced their listening skills, with one reporting, *‘… knowing when it’s not necessarily your turn to speak. Like, knowing when…you should listen, and then talk or just like, listen the whole time. …interviews like helped me with… regular human being skills’.* Lastly, some peer researchers shared the importance of learning time management skills. As explained by one participant, *‘…what I would give advice to is just make like a schedule in your mind. And like just plan things one by one like let’s say…I have to rest for…I’d say 30 minutes. And then by like, 5:30 I kind of practice…my skills for interviewing, then like, tomorrow morning, I have to go to work…when I come back, maybe I’ll practice more…’.*

#### Opportunity for open dialogue related to school health promotion

Participants shared their enjoyment of having the opportunity to discuss health and well-being issues with their peers, as well as learning from different perspectives. Participants commonly shared that this project provided a platform to discuss topics that are not regularly discussed in school. As stated in the words of the peer researchers, *‘…health and well-being because like, we don’t really like…nobody really talks about it’* and ‘*Especially like me and my friends, it was just never a topic that would…be brought up. But like hearing…what they had to say about that was…nice’.* Furthermore, peer researchers appreciated hearing different perspectives and opinions throughout the process. As stated by one peer researcher, *‘I learned that school experience is incredibly different for everyone, so I learned how to see other people’s perspectives and compare mine to find differences and similarities’.* Overall, the opportunity to conduct a research project opened peer-to-peer dialogue on the subject matter.

#### Support throughout project

Collectively, peer researchers voiced their overall enjoyment and support they received throughout the experience, such as stating, *‘I really liked how…nice everyone was, and how…helpful and…how everyone would… rephrase questions if I didn’t understand it’.* Peer researchers also enjoyed having the opportunity to do something that involved their friends, with one noting *‘I liked the interviewing part because I got to interview my friends*’. Challenges identified in the research project largely related to the interview questions being unclear or confusing, with peer researchers suggesting changes to the interview guide such as ‘*…some of the wording since some my friends…got confused a lot’ and ‘making the interview questions easier, they were difficult to understand’.* Furthermore, some peer researchers voiced how they would have to reword and explain questions in different ways to help the interviewees understand the question. Other points made included ensuring a suitable amount of time for movement and interaction during training activities as this was voiced as some of the most liked parts of training, with one participant expressing that, ‘*The best parts of the training were getting to play energizer games along with getting the recorders and instructions of how to interview peers’.*

## DISCUSSION

This article provides an overview of using a peer researcher method within a YPAR project to train youth to conduct interviews with their peers regarding students’ perspectives on school health promotion. It also outlines the evaluation results of the peer researchers’ experience with the second iteration of the YPAR project.

### Process of Youth Participatory Action Research

Our study provides an overview of the stages implemented to complete a YPAR project using a peer researcher method within the context of school health promotion, which comprised of: (i) peer researcher training, (ii) practicing, recruiting and conducting interviews and (iii) data interpretation workshop. Students were involved in various phases of the research including identifying concerns of interest, co-developing the data collection tool, conducting semi-structured interviews and interpreting the data analysis. The involvement of youth at these various stages of the work is consistent with other research using similar participatory approaches (Soleimanpour et al., 2008; Jardine & James, 2012; Lile & Richards, 2018). Furthermore, our project placed focus on building a supportive training environment for the peer researchers and adult facilitators—a critical component highlighted in the literature to help mitigate power differentials and build trust (Ozer et al., 2010; Jardine & James, 2012; Kim, 2016). However, YPAR often occurs on a spectrum with children and youth being involved to different degrees and intensities (Pan-Canadian Joint Consortium for School Health, 2018); therefore, the shape and form across research studies can differ. Our study prioritized involving students in the development and implementation of the data collection method, while other YPAR projects have focused on other stages of the process, such as data analysis (John-Akinola et al., 2013; Garnett et al., 2019). For example, John-Akinola et al. (2013) examined children’s views on their participation in schools using a participatory focus group involving a data analysis component where students played an adapted version of the card game ‘snap’ to pair sticky notes together and create themes from similar concepts and ideas. Although our project involved a data interpretation workshop, it was beyond our resources to train the peer researchers in analyzing the raw data of the interviews. Involving youth in data analysis is timely, and intentional planning and thought need to go into how to do this properly and collaboratively. Liebenberg et al. (2020) provide a great thought piece and example of how they used participatory thematic analysis within their project with Indigenous youth in Atlantic Canada to understand the importance of space and place. We hope that in future projects we can continue to push our work further by engaging youth even more meaningfully in the data analysis process.

Furthermore, the detailed description of the process used in this study is integral to understanding how a peer researcher method can be aligned with a YPAR approach. Beyond photovoice, a visual method often used in YPAR projects (Henry et al., 2013; Warne et al., 2013) that involves a formalized nine-step process (Wang & Burris, 1997), other participatory methods used with children and youth lack consistent techniques. As the peer researcher method is a relatively new technique in YPAR (Lile & Richards, 2018), and school health promotion alike, there is minimal research that has outlined this approach, specifically in the content area. Thus, this article provides an in-depth description of the peer researcher method used within YPAR to help fill this gap, as well as aid in the dependability and transferability of the research (Creswell & Poth, 2016). This detailed overview provides an in-practice example of how to embed YPAR conceptual principles of youth areas of interest, collaboration and transformation previously outlined by Rodríguez and Brown (2009).

### Benefits of YPAR

Providing clarity on different methods used within YPAR is important as there is growing evidence of the powerful impact that a YPAR approach can have at the individual, interpersonal and system level (Ozer, 2017; Anyon et al., 2018; Branquinho et al., 2020). In our case, we were able to capture the individual-level benefits of being involved in a YPAR project, as well as the value of the peer researcher method specifically from the perspectives of youth. Peer researchers shared that the project taught them new skills and content knowledge, as well as supported their personal development. This is in alignment with existing research (Powers & Tiffany, 2006; Anyon et al., 2018), including a recent systematic review on the outcomes, methods and future direction of YPAR in the USA, which concluded that participatory research with youth is associated with improvements in youth agency and leadership, academic or career endeavours, as well as social, interpersonal and cognitive skills (Anyon et al., 2018). Furthermore, the current study allowed youth to learn and understand new and different perspectives related to the topic of school health promotion and youth engagement from other peer researchers, as well as interviewees. Understanding and considering perspectives beyond their own is foundational for building critical social awareness—the connection of individual perspectives to larger social themes and issues (Lile & Richards, 2018).

Partnering with youth in research adds value to the scientific process by providing a level of credibility to the research that may be absent from traditional methods (Jennings et al., 2006). As youth are experts in their own realities, they can provide credibility to an array of methodological components (Hawke et al., 2018). Benefits of YPAR to the research process were readily outlined by Jardine and James (2012), project that worked with youth to examine tobacco use in schools in the Northwest Territories of Canada using photovoice and peer-to-peer interviews. This research outlined the value of YPAR in the scientific process indicating that the research was perceived to be more credible, and appropriate to the students, and that it provided a pedological opportunity for youth to gain research experience as well as a sense of ownership throughout the study. Within our study, the engagement of youth across the research process can be seen through the adaptation of interview questions and techniques, interpretation of the data analysis and mitigation of power dynamics by having youth rather than adult researchers interview participants. The latter is often promoted as a key benefit of the peer researcher process (Jardine & James, 2012; Lile & Richards, 2018; Nelson Ferguson et al., 2023), such that youth participants may feel more comfortable sharing their thoughts, feelings and perspectives with their peers over adult researchers.

### Strengths and limitations

There were various strengths to the YPAR process and evaluation that should be highlighted. First, we were able to recruit a diverse group of youth as peer researchers within the study. As YPAR is often promoted as a process to engage individuals and/or groups who are equity deserving, diversity in the participants engaged is a key asset to the project. Second, an array of strategies was used to mitigate power dynamics between adult team members and peer researchers, including dedicated time for capacity building, skill development and self-reflection at the forefront, holding the training outside of the school setting, diversity in facilitators to ensure there were adults who held similar identities to the peer researchers and compensating them for their time, and skills. Third, the peer researchers were part of the data analysis stage of the research process through the data interpretation workshop, a component that is often left out of participatory processes. We were also able to collect evaluation feedback through quantitative and qualitative methods, to elucidate the peer researchers’ experience and perspectives throughout the process.

Despite the strengths of this YPAR process and evaluation, there were limitations and learnings that need to be considered. The 2-day in-person training may not be enough time to build rapport within and across peer researchers and adult facilitators, or for all peer researchers to build the necessary competencies to conduct the interviews. Based on the findings, peer researchers may have benefitted from further support during stage 2—practicing, recruiting and conducting interviews to help clarify the interview guide questions. Future studies using a peer researcher method may consider adding a check-in point during stage 2 to provide the option for peer researchers to ask questions and receive support from adult team members. Lastly, the focus group/interviews, as well as the data interpretation workshop were held online rather than in-person, minimizing the amount of engagement and attendance from the peer researchers compared to an in-person event. There were multiple potential reasons for the lack of attendance at the data interpretation workshop such as the workshop being held a few months after the completion of the interviews, the timing fell on one of the hottest days of the Spring and an online workshop may have not been appealing to the peer researchers.

### Implications for policy and practice

By sharing the process of conducting a YPAR project and outlining the experience of the youth involved, we hope to raise awareness of the rights of, and need to, engage youth on issues and concerns that affect their lives. The study provides a detailed overview of how to engage youth in research in a genuine manner, to ensure the work has real-world transferability for educators and health professionals alike. Such activities seek to shift traditional power dynamics in our society, in recognition that shifting authority can help build professional and personal skills in youth. This work contributes to growing school health promotion efforts that build authentic strategies to engage students in decision-making. Meaningful engagement of students is a top priority for HPS initiatives locally and nationally, meaning our learnings can be applied to future HPS efforts.

## CONCLUSION

This article described the use of a YPAR approach where youth were trained as peer researchers to conduct interviews with their peers to learn about their perspectives on youth engagement and health promotion within the school setting. It also summarizes an evaluation conducted to capture the perspectives of youth who were involved in the YPAR project. Aligning with current literature, the evaluation confirmed individual-level benefits of a YPAR process including advancement of research skills, improvements in personal development and an increase in content-specific knowledge. This article further adds to a growing recognition of the need to authentically engage youth in matters that affect their lives, specifically in relation to school health promotion. YPAR is a valuable and useful approach to embedding youth engagement within a systematic research process.

## Supplementary Material

daad174_suppl_Supplementary_Document_1

daad174_suppl_Supplementary_Document_2

daad174_suppl_Supplementary_Document_3

daad174_suppl_Supplementary_Document_4

daad174_suppl_Supplementary_Document_5
