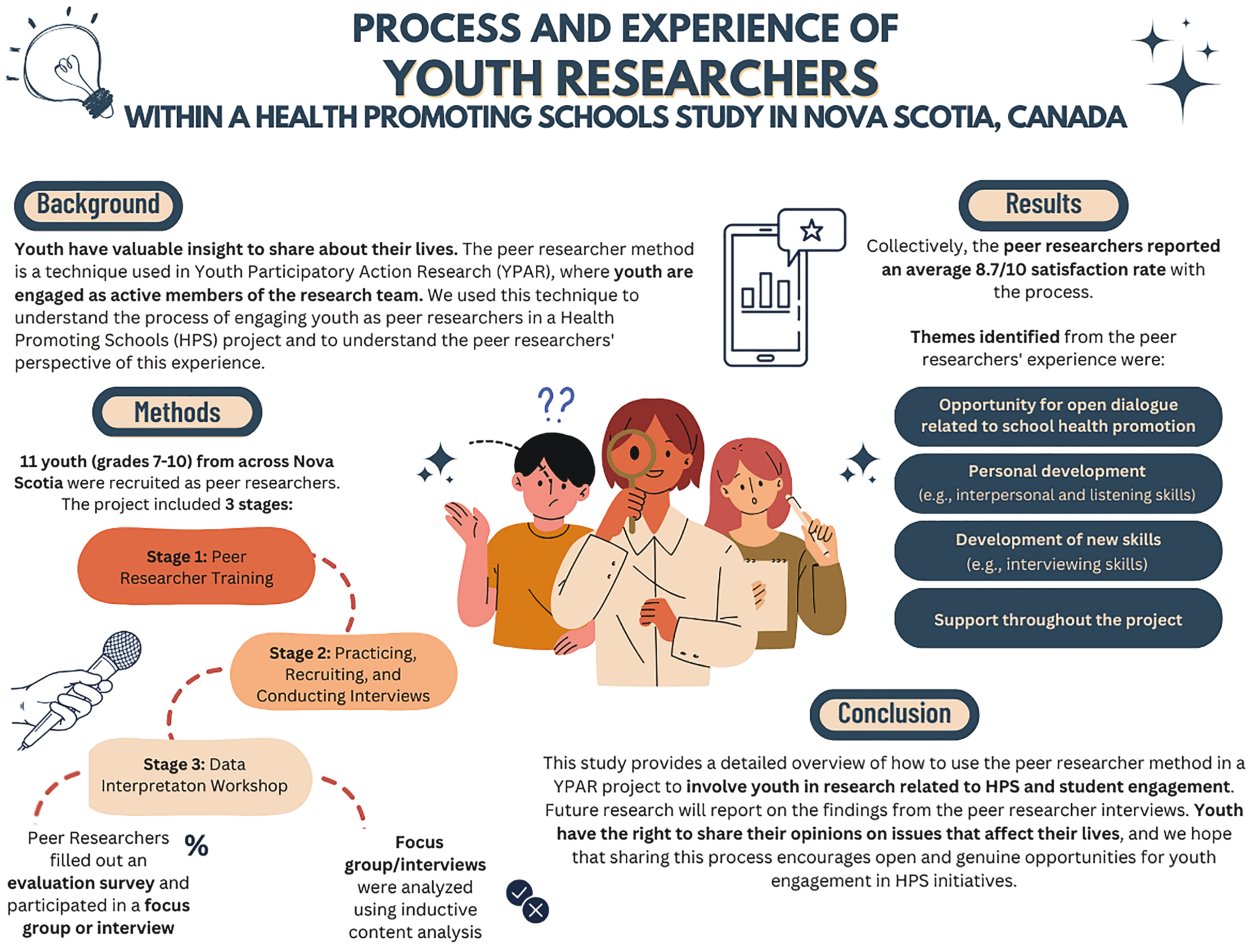# Correction to: Process and experience of youth researchers within a Health Promoting Schools study in Nova Scotia, Canada

**DOI:** 10.1093/heapro/daae016

**Published:** 2024-02-14

This is a correction to: Julia C Kontak, Hilary A T Caldwell, Rena Kulzycki, Camille L Hancock Friesen, Sara F L Kirk, Process and experience of youth researchers within a Health Promoting Schools study in Nova Scotia, Canada, Health Promotion International, Volume 38, Issue 6, December 2023, daad174, https://doi.org/10.1093/heapro/daad174

The originally published version of this manuscript has been updated to correct a typographical error in the name of author Rena Kulczycki.

The graphical abstract has been updated to correct the title to ‘Process and experience of youth researchers within a Health Promoting Schools study in Nova Scotia, Canada’, and to remove the text ‘Space for authors names’, which was included under the title in error.

Finally, the following sentence has been updated to correct the error in the in-text citation of Lumivero, 2023:

Open-ended data from the evaluation questionnaire were imported into Nvivo Qualitative Data Software, version 1.7.1 (Lumivero, n.d.) for analysis in conjunction with focus group and interview data.