# Supplementary material for: Process and experience of youth researchers within a Health Promoting Schools study in Nova Scotia, Canada
Source: Health Promot Int. 2023 Dec 20;38(6):daad174. doi: 10.1093/heapro/daad174 (PMC10733659; doi:10.1093/heapro/daad174)
Supplement: daad174_suppl_Supplementary_Document_1 [file daad174_suppl_supplementary_document_1.pdf]

Peer Researcher Training Workshop

Welcome to Peer Researcher Training! Below outlines the agenda for the next two days

Thursday, July 21st: 12 pm - 5 pm

Time	Purpose
12 - 12:15	Welcome
12:15 - 12:45	Introduction to the day and each other
12:45 - 1:30	Creating comfort, group connection and community agreements
1:30 – 2:15	Lunch
2:15 – 2:30	Energizer
2: 30 – 3:00	Learn and activity about school health and well-being
3:00 – 3:25	Learn and activity about system change
3:25 - 3:40	Break
3:40 - 4	Learn and example of youth as changemakers in the school
4 – 4:30	Introduction to interview questions
4:30 - 5	Closing/orientation to tomorrow

Friday, July 22nd: 9 am - 4 pm

Time	Purpose
9 – 9:30	Introduction to the day and welcome
9:30 – 10:30	Interview Training Presentation
10:30 – 11:30	Developing interview guide together
11:30 – 12:15	Lunch
12:15 – 12:30	Energizer
12:30 – 1	Getting comfortable with recorders
1:00 – 2:00	Interview practice with peers
2:00 – 2:15	Break
2:15 – 2:45	Debrief on interview questions and training
2:45 – 3:30	Next steps after training (timeline, timesheets, responsibilities)
3:15 – 3:30	Evaluation
3:30 – 4	Closeout
