# Supplementary figures and images for: Process and experience of youth researchers within a Health Promoting Schools study in Nova Scotia, Canada
Source: Health Promot Int. 2023 Dec 20;38(6):daad174. doi: 10.1093/heapro/daad174 (PMC10733659; doi:10.1093/heapro/daad174)

**Supplementary Document 2: Rose, Bud and Thorn Facilitation Activity**


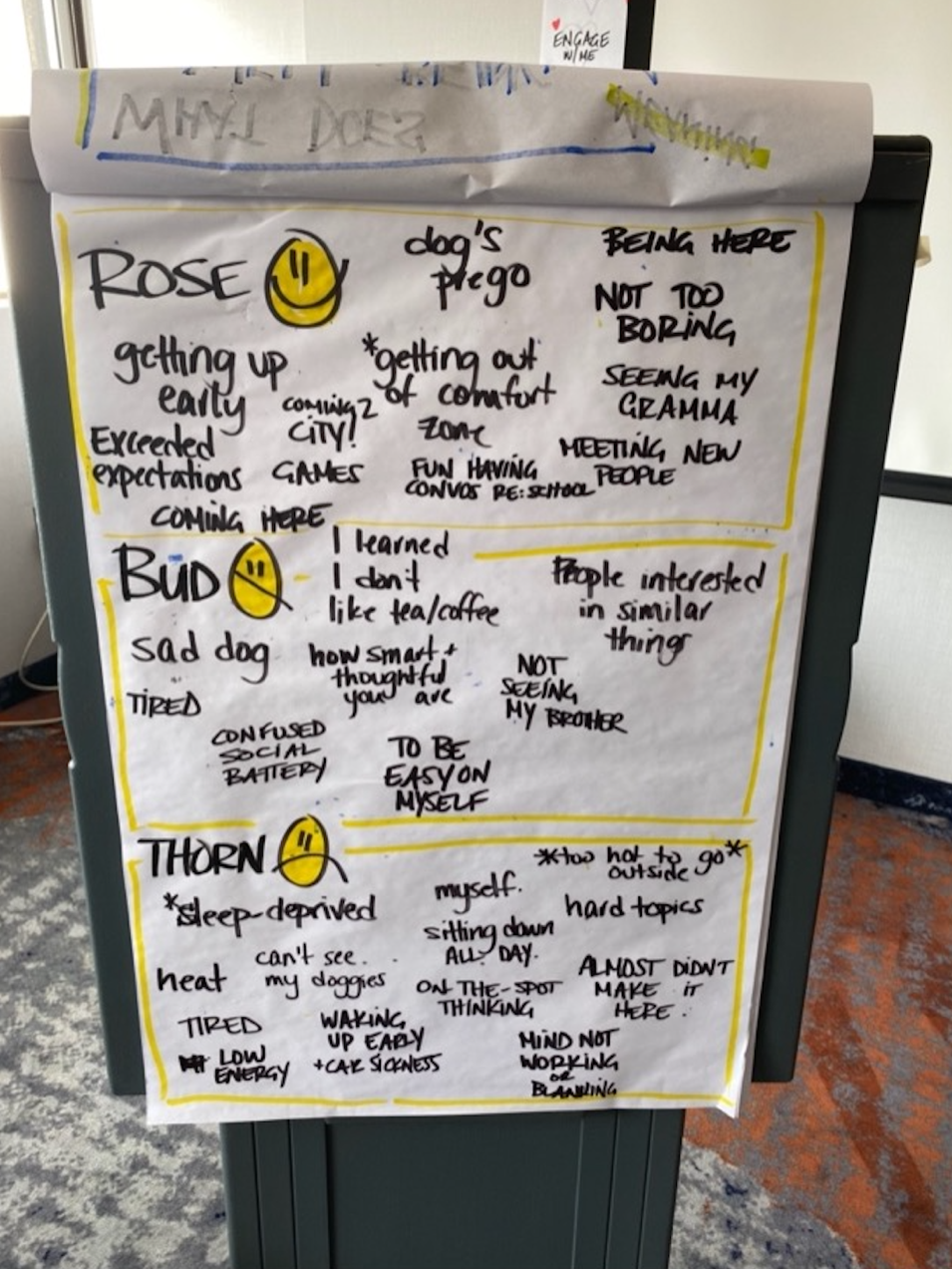

Supplement: daad174_suppl_Supplementary_Document_2 [file daad174_suppl_supplementary_document_2.docx]

**Supplementary Document 3: Example of School Health Promotion Interactive Activity**


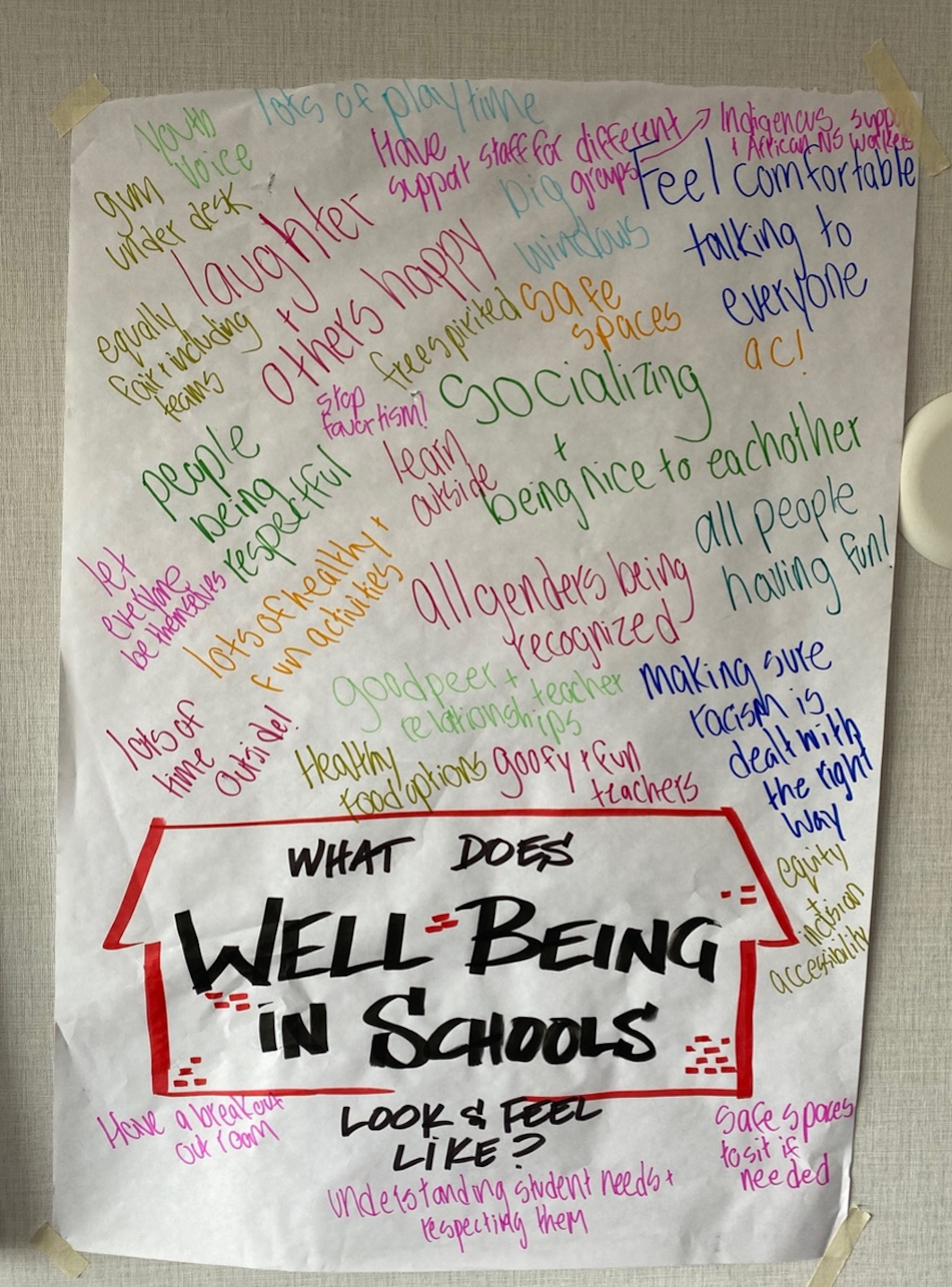

Supplement: daad174_suppl_Supplementary_Document_3 [file daad174_suppl_supplementary_document_3.docx]
