# Supplementary material for: Process and experience of youth researchers within a Health Promoting Schools study in Nova Scotia, Canada
Source: Health Promot Int. 2023 Dec 20;38(6):daad174. doi: 10.1093/heapro/daad174 (PMC10733659; doi:10.1093/heapro/daad174)
Supplement: daad174_suppl_Supplementary_Document_4 [file daad174_suppl_supplementary_document_4.docx]

**Supplementary Document 4: Peer Researcher Evaluation Questionnaire**

**Peer Researcher Training Survey**

1. Please indicate how much you agree or disagree with each statement

|  | **Strongly Disagree** | **Disagree** | **Neutral** | **Agree** | **Strongly Agree** | **Not Applicable** |
| --- | --- | --- | --- | --- | --- | --- |
| I felt prepared to attend the peer researcher training |  |  |  |  |  |  |
| I have a good understanding of what Health Promoting Schools means |  |  |  |  |  |  |
| The training was engaging and interactive |  |  |  |  |  |  |
| The training taught me about research ethics |  |  |  |  |  |  |
| I feel prepared to interview my peers |  |  |  |  |  |  |
| I am confident to interview my peers |  |  |  |  |  |  |
| I am excited to interview my peers |  |  |  |  |  |  |
| I felt supported during the training |  |  |  |  |  |  |
| I am interested in being a peer researcher with other projects |  |  |  |  |  |  |

1. What were the best parts of the training and why?
2. What were the worst parts of the training and why?
3. What is the most important thing you learned in your peer researcher training?

|  | **1**  **Low** | **2** | **3** | **4** | **5** | **6** | **7** | **8** | **9** | **10**  **High** |
| --- | --- | --- | --- | --- | --- | --- | --- | --- | --- | --- |
| On a scale of 1 to 10, please rate your overall satisfaction with the training |  |  |  |  |  |  |  |  |  |  |
