# Supplementary material for: Process and experience of youth researchers within a Health Promoting Schools study in Nova Scotia, Canada
Source: Health Promot Int. 2023 Dec 20;38(6):daad174. doi: 10.1093/heapro/daad174 (PMC10733659; doi:10.1093/heapro/daad174)
Supplement: daad174_suppl_Supplementary_Document_5 [file daad174_suppl_supplementary_document_5.docx]

**Supplementary Document 5: Semi-structured Questions for Focus group/interviews with Peer Researchers**

| **Questions related to Peer Researcher Experience** |
| --- |
| 1. What did you like best about the project and why?  - *Prompts: In-person training, learning how to interview, interviewing peers* |
| 1. What was helpful for you, and why?  - *Prompt: Did you feel supported by UpLift as a peer researcher? Why or why not?* |
| 1. What would you change/improve, and why?  - *Prompt: How could we have better supported you as a peer researcher?* |
| 1. What did you learn from the project? |
| 1. How can you apply this learning in the future? |
